# Supplementary material for: Splice-Junction-Based Mapping of Alternative Isoforms in the Human Proteome
Source: Cell Rep. Author manuscript; Available in PMC 2020 Jan 15. (PMC6961840; doi:10.1016/j.celrep.2019.11.026)
Supplement: 2 [file NIHMS1546469-supplement-2.zip › DF1/PXD009737/TestisTrypsin/TestisTrypsin_77_SH3GLB1_LEGDNIMSEQELRIT.pdf]

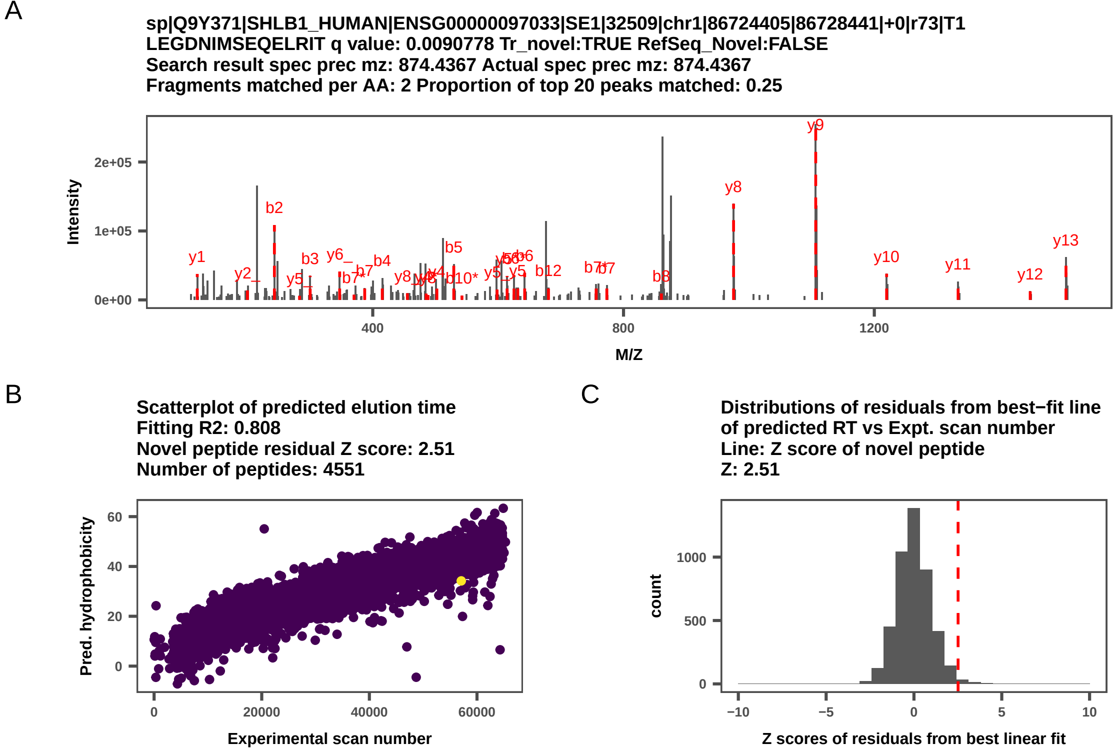
A
sp|Q9Y371|SHLB1_HUMAN|ENSG00000097033|SE1|32509|chr1|86724405|86728441|+0|r73|T1
LEGDNIMSEQELRIT q value: 0.0090778 Tr_novel:TRUE RefSeq_Novel:FALSE
Search result spec prec mz: 874.4367 Actual spec prec mz: 874.4367
Fragments matched per AA: 2 Proportion of top 20 peaks matched: 0.25
Intensity
M/Z
B
Scatterplot of predicted elution time
Fitting R2: 0.808
Novel peptide residual Z score: 2.51
Number of peptides: 4551
Pred. hydrophobicity
Experimental scan number
C
Distributions of residuals from best−fit line
of predicted RT vs Expt. scan number
Line: Z score of novel peptide
Z: 2.51
count
Z scores of residuals from best linear fit
